# The Use of Systematic Clinical Approach in Diagnosing Rare Cases of Secondary Hypertension: A Case Report of Mid Aortic Syndrome in a Young Patient

**DOI:** 10.7759/cureus.19215

**Published:** 2021-11-02

**Authors:** Mohammed Al-Musawi, Akeel Yuser, Suhad AlOmaishi, Oula Kareem, David Rubay

**Affiliations:** 1 Surgery, Anschutz Medical Campus, University of Colorado, Aurora, USA; 2 Cardiovascular Surgery, Ibn AlNafees Center for Thoracic and Cardiovascular Surgery, Baghdad, IRQ; 3 Internal Medicine, Life Alliance Organ Recovery Agency, University of Miami, Miami, USA; 4 Department of Surgery, University of Colorado, Aurora, USA; 5 Trauma and Surgical Critical Care, University of Florida College of Medicine, Gainesville, USA

**Keywords:** mid aortic, giant cell arteritis, takayasu arteritis, fibromuscular dysplasia, retroperitoneal fibrosis

## Abstract

Mid aortic syndrome (MAS) is a rare disease that occurs in children and young adults. The most important clinical feature reflecting vascular involvement is the presence of systemic hypertension. The diagnosis is usually made during the imaging assessment of secondary hypertension when routine echocardiography fails to identify the characteristic morphological or Doppler flow patterns associated with thoracic arch coarctation in the presence of the clinical features of aortic vascular obstruction. In this report, we present a case of a 22-year-old male who presented with systemic hypertension not responding to medical treatment, and whose systematic diagnostic workup revealed the diagnosis of MAS involving both renal arteries.

## Introduction

Mid aortic syndrome (MAS) is a rare disease that presents in children and young adults and constitutes 0.5-2% of all cases of aortic narrowing [1]. MAS is a term used to describe the localized narrowing of the distal thoracic/abdominal aorta regardless of etiology [2].

Although MAS was first described almost 60 years ago, its etiology remains unknown; its pathogenesis is mainly speculative, and most cases of MAS are idiopathic. Some cases have been described in association with genetic and acquired diseases [3]. Congenital narrowing has been thought to occur due to the over-fusion of embryonic dorsal aortas during the fourth week of gestation [2]. Acquired causes include different types of vasculitis such as giant cell arteritis, Takayasu arteritis, or even other conditions like fibromuscular dysplasia (FMD), neurofibromatosis, atherosclerosis, retroperitoneal fibrosis, and mucopolysaccharidoses [3]. The most significant clinical feature reflecting vascular involvement is systemic hypertension (>90% of cases), with claudication and ischemic intestinal symptoms less frequently observed [4]. Renovascular disease is one of the most common causes of secondary hypertension. It is most commonly attributed (85% of cases) to atherosclerotic renal artery stenosis (RAS) and less frequently to causes like FMD, arterial occlusion from embolic disease (iatrogenic following endovascular aortic stents and grafts), aortic dissection, and vascular inflammatory diseases such as Takayasu arteritis, vasculitis, and scleroderma [5]. The treatment of secondary hypertension is appealing to physicians, as providing the appropriate therapy can be curative and profoundly impacts cardiovascular outcomes and quality of life. However, despite the potential benefits of early diagnosis and treatment of patients, many cases of secondary hypertension remain unfortunately undiagnosed [6]. Widespread testing of all hypertensive patients is not cost-effective and frequently leads to false-positive results; hence, the selective screening of patients both at high risk for or with signs and symptoms suggestive of secondary hypertension is necessary, with the classic presentations that trigger screening for secondary hypertension including resistant or refractory hypertension [7]. Rapid changes in blood pressure in patients younger than 30 or older than 50 years in age, or accelerated hypertension in those already treated for hypertension, are clues for diagnosing renovascular hypertension [8]. Renal ultrasound with Doppler of the renal arteries is the initial imaging technique to diagnose RAS [9]. CT and magnetic resonance angiography (MRA) effectively establish the presence of RAS with a sensitivity and specificity above 90% and are often helpful when intervention is for procedural planning [8].

The diagnosis is usually made during the routine imaging assessment for hypertension when routine echocardiography fails to identify the characteristic morphological or Doppler flow patterns associated with thoracic arch coarctation in the presence of the clinical features of aortic vascular obstruction. This constellation prompts additional angiographic or cross-sectional imaging like CT angiography (CTA) or MRA, allowing for the characterization of the vessel wall [10]. RAS at initial presentation is common (66%), and approximately 60% of these cases have bilateral RAS, while 20% have unilateral renal artery involvement [1]. The goal of treatment is to normalize the blood pressure, avoid any clinical symptoms or complications of hypertension, and preserve the renal function as much as possible. Treatment options include medication, percutaneous transluminal renal angioplasty (PTRA) and stent implantation, surgical revascularization, and unilateral nephrectomy in some difficult cases [2,11]. However, in many cases, adequate blood pressure control cannot be achieved despite providing the best medical therapy without interventional relief of the associated aortic or renovascular stenoses. This adequate blood pressure control is mostly achieved by combining surgical aortic reconstruction with revascularization of the kidneys [4]. The extensive nature of the aortic and renal lesions in MAS generally reduces the endovascular success rate compared to those with an isolated RAS [11]. The absolute indications for surgery include poor BP control with high doses of antihypertensive agents, evidence of end-organ damage [left ventricular hypertrophy (LVH) and hypertensive retinopathy], and evidence of the deterioration in the renal function and loss of renal mass [12].

## Case presentation

**Figure 1:**
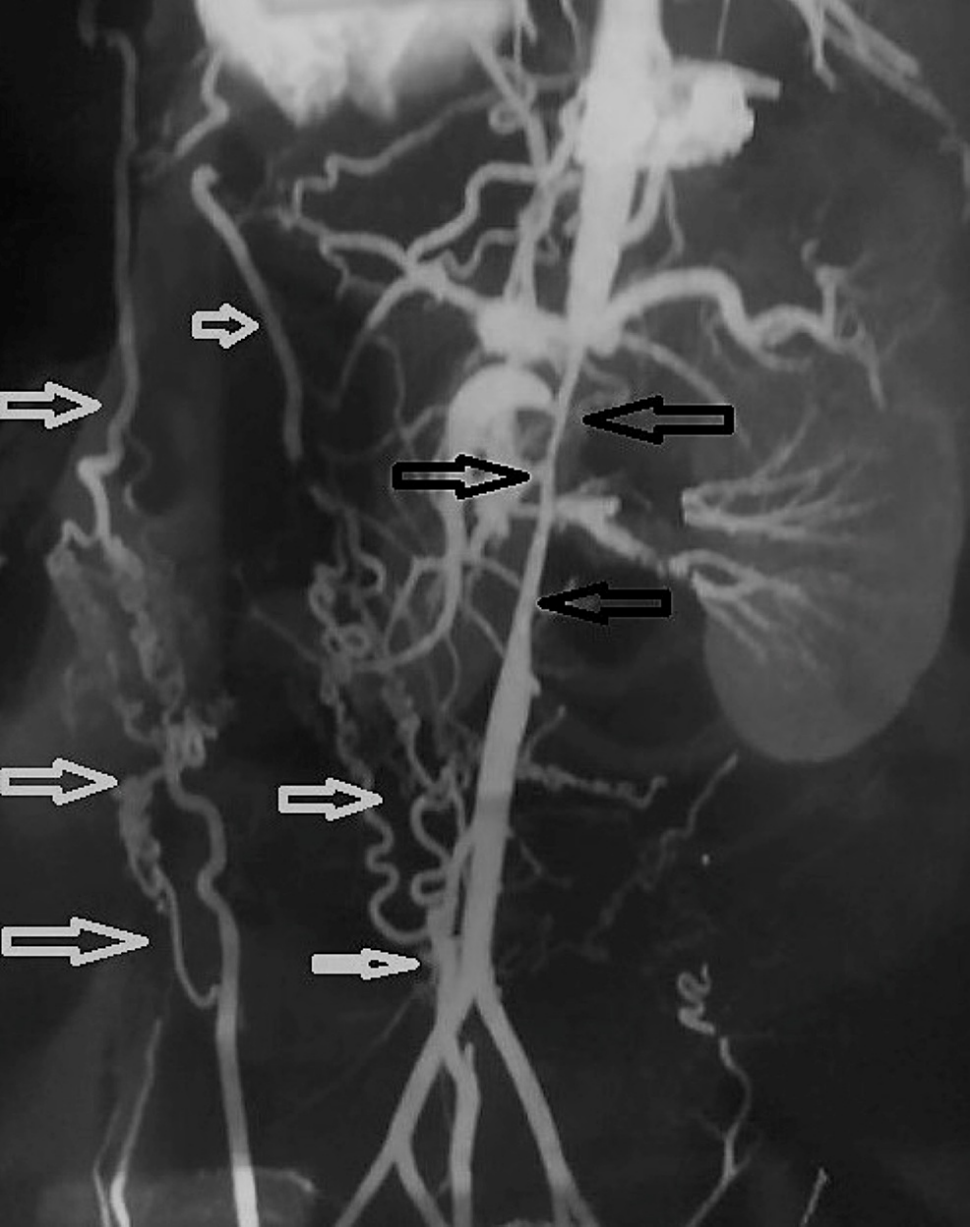
CTA image 1 The image shows the narrowing of abdominal aorta distal to celiac artery and superior mesenteric artery and proximal to the inferior mesenteric artery (black arrows), and the internal thoracic artery-inferior epigastric artery collateral is one of the collaterals supplying blood flow to the lower extremity CTA: computed tomography angiography

**Figure 2:**
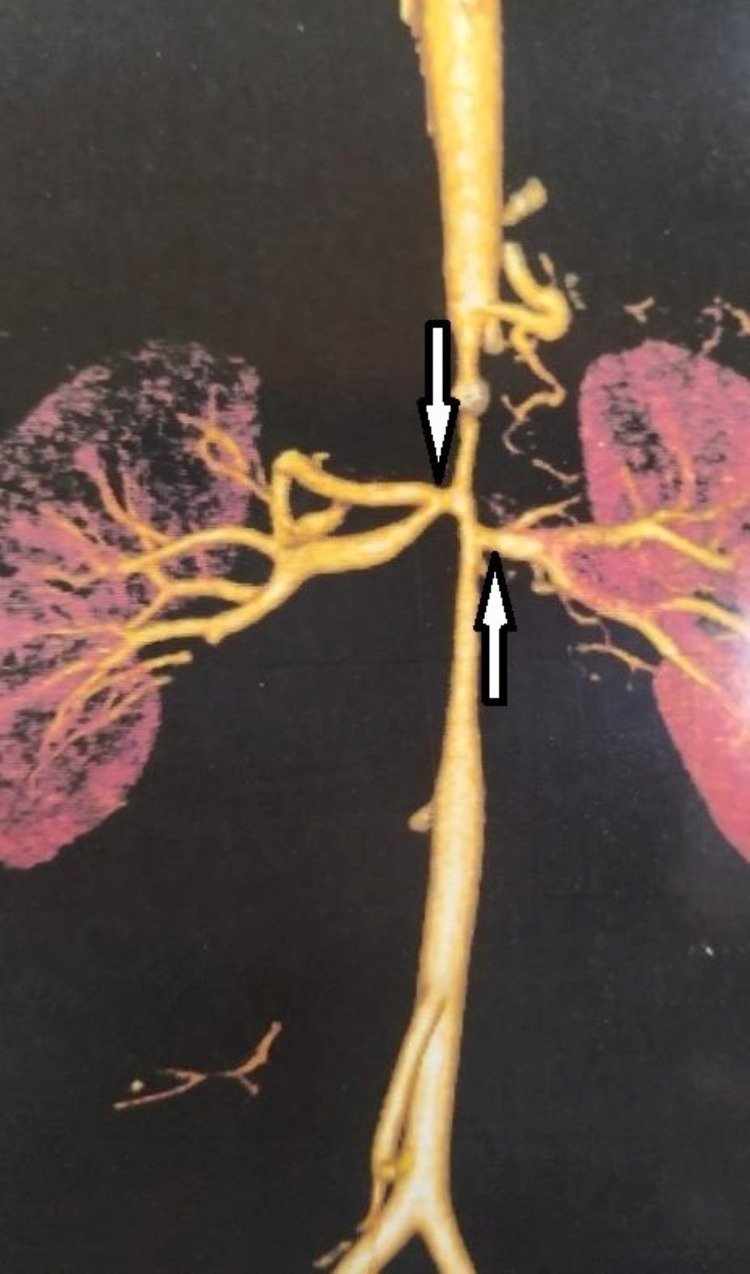
CTA image 2 The image showing ostial narrowing of the renal artery CTA: computed tomography angiography

The patient was a 22-year-old male who had been referred to our heart center with symptoms of headache, easy fatigability, lower limb claudication, and mild elevation of renal indices. The patient had high arterial blood pressure and was on high-dose triple antihypertensive drug therapy (angiotensin-converting enzyme inhibitor, calcium channel blocker, and thiazide diuretic) without any significant clinical response. On clinical examination, the patient had diminished femoral arterial pulses bilaterally. The patient’s young age and the lack of any significant response to a combination of antihypertensives and the slightly elevated renal indices led us to look for secondary causes for the elevated blood pressure. When transthoracic echocardiography (TTE) with color Doppler returned negative for coarctation, it showed LVH. We decided to send the patient for renal duplex studies, which showed significant narrowing of the abdominal aorta and bilateral ostial renal arteries. The next step was to have the patient undergo a CTA of the thoracic and abdominal aorta. This time, the result revealed a diagnosis of MAS showing the narrowing of abdominal aorta distal to celiac artery and superior mesenteric artery and proximal to the inferior mesenteric artery (Figures 1, 2). It also showed that the internal thoracic artery-inferior epigastric artery (ITA-IEA) collateral was one of the collaterals supplying blood flow to the lower extremity (Figure 1), which is expected in these cases.

## Discussion

MAS is a rare clinical condition with no characteristic presentation. Still, this syndrome can lead to severe hypertension, diminished or absent femoral pulses, lower extremity claudication, and audible arterial bruits. Laboratory analysis can show nonspecific elevation of inflammatory markers such as erythrocyte sedimentation rate (ESR) and C-reactive protein (CRP) [13]. Although MAS has been observed in infants, it often occurs in children over five years of age, and the mean age at diagnosis is about 20 years [11]. The list of secondary causes of hypertension in young age groups is well known. Still, we need to tailor our differential diagnosis based on the clinical findings without going through the whole list of investigations [14]. The clues in our case for an occlusive vascular pathology were bilateral weak femoral artery pulses, and the claudication is the main finding in these cases [15]. Nevertheless, it was unclear if it was coarctation of the aorta, which is relatively more common, or another aorto-occlusive disease [1,6,16,17]. Hence, we started looking for the more common thing: the coarctation of the aorta through using TTE, which came back negative. Our next step was to assess renal artery occlusive disease through renal duplex studies, which suggested a narrowing of the abdominal aorta and bilateral ostial renal arteries. Based on this result, we looked for the anatomy of the thoracoabdominal aorta via CT with contrast (CTA) to assess the extent of the occlusive pathology. This time, the diagnosis was precise with the full anatomic display of the location and the extent of the lesion, and the patient was diagnosed with MAS.

The patient was referred to the division of vascular surgery, given the complexity, the length of the lesion, a poor BP control with high doses of antihypertensive agents, and evidence of end-organ damage (LVH) and deterioration in the renal function. This led to a decision to perform aorta-aortic bypass graft and bilateral renal artery bypass grafting rather than endovascular dilation and stenting of the abdominal aorta and bilateral renal arteries [11,12]. The extensive nature of the aortic and renal lesions in MAS generally reduces the endovascular success rate compared to those with an isolated RAS [11]. The absolute indications for surgery include poor BP control with high doses of antihypertensive agents, evidence of end-organ damage (LVH and hypertensive retinopathy), and evidence of the deterioration in the renal function and loss of renal mass. The relative indications for surgery include poor compliance with the medication regime, and if the patient has reached an age where only a single-stage definite operation should be required [12].

## Conclusions

MAS is a rare disease in children and young adults, which presents as secondary arterial hypertension. A systematic clinical approach can narrow down the differential diagnosis and subsequently lead to the use of the appropriate diagnostic tool. Open surgery and endovascular approach are commonly employed for managing this pathology, and the management should be tailored according to the availability of the resources, extent of the pathology, and associated comorbidities.
